# CDiP technology for reverse engineering of sporadic Alzheimer’s disease

**DOI:** 10.1038/s10038-022-01047-8

**Published:** 2022-06-10

**Authors:** Takayuki Kondo, Yuichiro Yada, Takeshi Ikeuchi, Haruhisa Inoue

**Affiliations:** 1 Center for iPS Cell Research and Application (CiRA), Kyoto University, Kyoto, Japan; 2 Medical-risk Avoidance based on iPS Cells Team, RIKEN Center for Advanced Intelligence Project (AIP), Kyoto, Japan; 3 iPSC-based Drug Discovery and Development Team, RIKEN BioResource Research Center (BRC), Kyoto, Japan; 4 Department of Molecular Genetics, Brain Research Institute, Niigata University, Niigata, Japan; 5 Institute for Advancement of Clinical and Translational Science (iACT), Kyoto University Hospital, Kyoto, Japan

**Keywords:** Alzheimer's disease, Genome-wide association studies, Induced pluripotent stem cells

## Abstract

Alzheimer’s disease (AD) is a neurodegenerative disease that causes cognitive impairment for which neither treatable nor preventable approaches have been confirmed. Although genetic factors are considered to contribute to sporadic AD, for the majority of AD patients, the exact causes of AD aren’t fully understood. For AD genetics, we developed cellular dissection of polygenicity (CDiP) technology to identify the smallest unit of AD, i.e., genetic factors at a cellular level. By CDiP, we found potential therapeutic targets, a rare variant for disease stratification, and polygenes to predict real-world AD by using the real-world data of AD cohort studies (Alzheimer’s Disease Neuroimaging Initiative: ADNI and Japanese Alzheimer’s Disease Neuroimaging Initiative: J-ADNI). In this review, we describe the components and results of CDiP in AD, induced pluripotent stem cell (iPSC) cohort, a cell genome-wide association study (cell GWAS), and machine learning. And finally, we discuss the future perspectives of CDiP technology for reverse engineering of sporadic AD toward AD eradication.

## Introduction

Neurodegenerative disorder is a pathological condition in which specific cell types gradually degenerate, leading to the disruption of their functions and networks. Among the neurodegenerative diseases, Alzheimer’s disease (AD) is the most common neurodegenerative disorder, accounting for 60–80% of all dementia cases [1]. There are over 50 million people worldwide living with AD or dementia, and this number is estimated to exceed 100 million by 2050 [2]. With recent developments in biology and genetics, the understanding of various molecular pathologies of dementia has progressed, but at present, there are only limited anti-symptomatic treatments for AD, and there is no curative treatment. To develop therapies for neurodegenerative diseases, it is necessary to have a deep understanding of the causes and their pathophysiology. Among the risk factors for AD, aging, biological gender, and genetic differences provide the strongest evidence [1, 3].

The search for the genetic background of AD was initially advanced by the genetic analysis of large pedigrees with familial AD (FAD). This investigation of FAD revealed mutations in *APP*, *PSEN1*, and *PSEN2* genes, which cause FAD with an autosomal dominant inheritance pattern. On the other hand, *APOE* genotype was identified as a genetic background of sporadic AD (SAD), which accounts for 90–95% of all AD cases [4]. Since 2002, genetic case-control studies, called genome-wide association studies (GWAS), have been developed to identify AD-relevant genotypes. In addition, methods such as whole exome sequencing (WES) and whole genome sequencing (WGS) with larger scales of AD cohorts have been utilized to identify greater numbers of rarer variants [1]. Thus, as the population size of the genetic cohorts grows, the genetic background of AD, which occurs more rarely, has become elucidated. To clarify the relationship between these genotypes and AD pathologies, researchers accumulated experimental results by introducing identified AD-related genes into cell lines and animal models. Such a model has been successful in reproducing one aspect of FAD. However, when introducing AD-related genes into cell-lines or animal models, the amounts of induced genes were several tens of times comparing with that of the physiological state in AD patients. Therefore, there are limitations to applying these cell lines or animal models to the evaluation of compounds or therapies to improve the AD pathology, which originated from the physiological expression patterns of AD-related genes. In fact, anti-AD therapeutics that have been very successful in rodent models have shown little clinical benefit in humans. This disappointing fact highlighted the additional importance of considering the cellular characteristics of human beings and the genetic background of patients for elucidation of the pathogenic mechanisms of AD.

To understand the genetic background of AD patients, we utilized the induced pluripotent stem cell (iPSC) technology born in 2006 [5, 6] and established patient-cell-based models of AD [7]. In this section, we reviewed the AD models based on iPSCs to elucidate the genetic involvement of AD pathology and introduced a novel concept to understand the genetic background of SAD cases as well as future perspectives.

## iPSCs provide various kinds of cell types

**Fig. 1:**
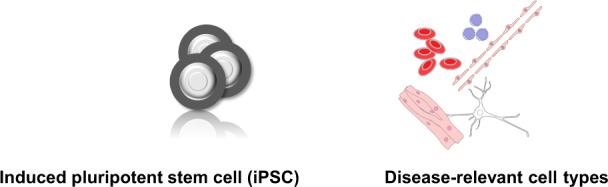
iPSCs can be differentiated into various kinds of disease-relevant cell types

Since 2007, reprogramming technology has made it possible to induce human pluripotent stem cells from somatic cells, thereby establishing iPSCs. iPSC technology has revolutionized disease research and personalized medicine [8, 9]. iPSCs established from patients can be differentiated into various cell types while inheriting all the genetic information of the patients. The brain is composed of not only neurons but also various cell types including astrocytes, oligodendrocytes, microglia, and vascular endothelial cells (Fig. 1). To recapitulate the human brain pathology, methods for producing various cell types of the brain have been developed over recent decades. Neuropathological analyses and research using AD models have suggested that there are diverse pathologies for each cell type [10–13]. Based on these facts, iPSCs are the resources of the cell types in relation to each pathophysiology of AD. These characters of iPSCs provide opportunities to establish disease models to allow understanding of the genetic background of AD patients.

## iPSC research for familial Alzheimer’s disease

Amyloid plaque and neurofibrillary tangles, which are the central neuropathological changes in AD, are known to contain amyloid β (Aβ) protein and tau protein as major constituents, respectively. Genetic linkage analysis of FAD identified Aβ precursor protein (*APP*), presenilin 1 (*PSEN1*), and presenilin 2 (*PSEN2*) as its causative genes. These FAD-creating genes were involved in the Aβ production pathway, and mutations in *APP*, *PSEN1*, and *PSEN2* alter the Aβ production [14]. Therefore, the amyloid cascade hypothesis that Aβ protein aggregation occurs first, eventually leading to neuronal cell death, has been widely accepted [15]. This hypothesis is supported by clinical epidemiological studies and large-scale genetic studies [1, 16].

In the iPSC-based models of FAD with mutations in *APP*, *PSEN1,* or *PSEN2* [17–22], cortical neurons derived from FAD iPSCs exhibited more prominent Aβ or tau pathology compared to healthy control neurons. These researches provided the basis for proving that AD-related phenotypes can be modeled using neurons derived from the patient’s iPSCs. In addition, cortical neurons derived from some SAD iPSCs also exhibited pathological phenotypes such as increased levels in phosphorylated tau or endoplasmic reticulum stress, and also compound-responsiveness similar to those derived from FAD iPSCs [17, 18]. These observations suggest that iPSC-derived neurons can model different pathological conditions and drug responsiveness differences in individual patients. Furthermore, iPSC models had been adopted to evaluation or screening assays to identify compounds that can improve AD phenotypes [23–27].

Screening with patient iPSCs provides information regarding candidate compounds and therapeutics for AD. However, since the genetic background of AD patients is diverse, a strategy to estimate the kind of AD population that can show the effectiveness of candidates is desired. Therefore, we administered therapeutic candidate compounds to cortical neurons that originated from iPSCs of FAD and SAD patients with different gene mutations, or from healthy individuals. Also, previously, “in vitro trials” had already been conducted to predict the result of a future clinical trial in a culture dish [23]. Such attempts are expected to lead to new medical treatments with expected therapeutic efficacy using iPSCs with patient genomic information. After these investigations, a Phase I/II clinical trial with the use of bromocriptine, identified by phenotypic screening of iPSC-derived neurons, was conducted to evaluate its safety and efficacy in FAD patients with *PSEN1* mutation, who had already been strong drug-responders in the previous “in vitro trial” [28].

## iPSC research for sporadic Alzheimer’s disease

The strongest genetic risk for SAD, which constitutes the majority of AD, is the apolipoprotein E (*APOE*) genotype. *APOE* was first identified as the susceptibility gene for late-onset AD in 1993. Differences in APOE genotypes are known to alter the structure of *APOE*, leading to AD risk in addition to affecting the lipid metabolism and cardiovascular function. Multiple studies have established iPSC-based models with different *APOE* genotypes or *APOE*-knockout clones, and succeeded in recapitulating the neuronal or glial phenotypes of AD [27, 29–32]. In addition to APOE genotypes, genetic cohorts such as GWAS have identified single nucleotide polymorphisms (SNPs) associated with the onset of AD. The iPSC-based system also succeeded in modeling the Aβ phenotypes that showed different responsiveness to brain-derived neurotrophic factor among different SNP genotypes in Sortilin Related Receptor 1 (*SORL1*) gene [33]. These studies proved that iPSC-based models can recapitulate the phenotypes originating from SNPs by harnessing SNP genotypes to AD phenotypes. However, these approaches still could not address the genome-wide analysis in a disease-relevant cell type.

## iPSC cohort of sporadic Alzheimer’s disease to conduct cell GWAS

The iPSC-based models of AD have become useful research tools for estimating the effects of various single genotypes on AD-relevant phenotypes. In particular, the combination of CRISPR–Cas9 genome editing and iPSCs enables us to prepare genetically identical sets of iPSCs other than the target genotype [34]. However, it will take vast amounts of time and effort to investigate the effects of various genotypes one-by-one. In addition, SAD has been considered as a polygenic disease in which thousands of genotypes contribute to the onset [1, 35, 36]. For these reasons, it is difficult to prepare isogenic iPSCs for all the different genotypes related to AD as identified in large-scale genetic cohorts. To solve this issue, it is necessary to prepare the population-scale datasets of iPSCs for a data-driven approach targeting AD-relevant genes, instead of for a candidate approach.

A sufficient amount of data is required to conduct data-driven approaches using iPSC. In order to perform a genome-wide gene investigation, it is necessary to prepare population-scale patient data. Therefore, we tackled this issue by establishing iPSCs from more than one hundred AD patients, and defined these iPSCs as the “iPSC cohort” [37] (Fig. 3). Established iPSCs were differentiated into cortical neurons, which retain similar differentiation characteristics among different patients, and they were expected to exhibit pathological phenotypes that reflect the genetic background of each patient.

When trying to explore the genetic background of AD, the pathology of AD is complex and biased by lifestyle and comorbidities. In particular, when researchers tried to investigate genes involved in the pathophysiology of AD such as Aβ production, they could not be analyzed by directly linking them to genes without bias. On the other hand, although there is a continuing discussion [38–40], most epigenetic modifications are reset during the reprogramming process from somatic cells to iPSCs. iPSCs show the epigenetic status, which is a similar status to the time point of the fertilized egg [41], and may reflect genomic information most directly without any bias such as from lifestyle and comorbidities. This property of iPSCs must enable us to reveal the direct link between AD pathological findings in the real world and the genome, which was previously overlooked in the clinical cohorts due to confounding factors.

**Fig. 2:**
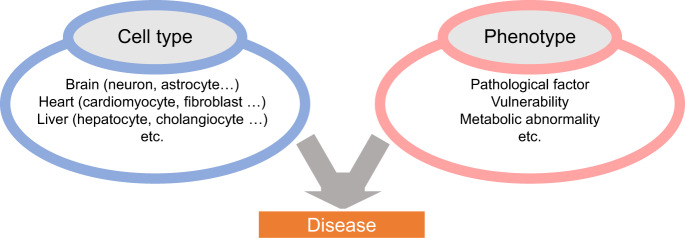
Dissecting the complexity of Alzheimer’s pathology into combination of cell types and phenotypes

**Fig. 3:**
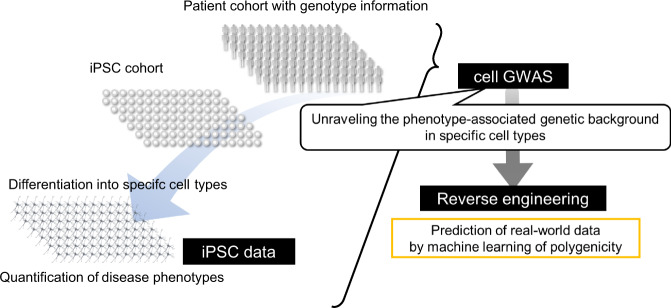
Schema of “Cellular dissection of polygenicity (CDiP)” technology for reverse engineering of sporadic AD

The brain is composed of a wide variety of cell types, and the genome is expected to affect different AD pathological findings depending on the respective cell types [10–13]. In order to dissect the complicated AD pathology, it would be useful to conduct analysis according to cell type separately (Fig. 2). iPSCs can be differentiated into various cell types that construct the brain through differentiation processes. Therefore, AD patient iPSCs can provide opportunities to analyze the AD pathological findings in a specific cell type. In this way, we proposed a concept to factorize the complex AD brain pathology into a combination of cell type and related AD phenotypes. Then, we set patient parameter variables, in other words “traits”, for each combination of cell type and phenotype, performed GWAS analysis, and named this scheme “cell GWAS” [37](Fig. 3).

As an example of cell GWAS, we prepared iPSC cohorts derived from 102 SAD patients who met the diagnostic criteria of AD. Cerebral cortical neurons were prepared from an iPSC cohort as a cell type, and cell GWAS was performed with Aβ production metabolisms as a phenotype. As a result, we identified 24 genetic loci associated with Aβ42/40 ratio. Of these, 5 genes have been reported to be associated with Aβ production, and 8 genes have been identified as AD-relevant genotypes in clinical GWAS. Therefore, cell GWAS partially overlaps with the results of clinical GWAS that requires a large patient population, and it is considered that genes related to the Aβ42/40 ratio in neurons are properly extracted. The remaining 11 were identified as newly identified AD-related genes. The reason why these genes could not be found until now is probably because various confounding factors may become noise during clinical GWAS alone. In addition, we confirmed that the Aβ42/40 ratio was changed when several genes found in cell GWAS were knocked down in cortical neurons derived from iPSCs of AD patients. Thus, cell GWAS using iPSC cohort has been shown to have the potential to discover new disease-related genes. In the future, the individual genes found in cell GWAS can be expected to be applied as therapeutic targets and diagnostic markers. In fact, we could identify rare variants in *KCNMA1* gene, as a risk for AD in two different independent genetic cohorts in studies conducted in North America and Japan, the Alzheimer’s Disease Neuroimaging Initiative (ADNI) cohort and the Japanese ADNI cohort. Genetic investigation of rare variants, indexed by whether AD develops, is one of the most powerful tools for exploring the missing heritability of AD. Because of the very low frequency of rare variants, a large sample size is required to identify the rare variants associated with AD. On the other hand, cell GWAS can perform analysis with high statistical power by using continuous variables for traits and preparing cell types related to pathological conditions. Therefore, even a small cohort of about 100 participants can reveal a genetic background that has been overlooked until now. In addition, using cell GWAS that limits the number of genes for the investigation of rare variants has the advantage of improving the statistical power of rare variant searches. In the future, it is expected that clinical analyses based-on WES and WGS and cell GWAS based on the iPSC cohort will cooperate with each other to clarify the genetic background as well as the pathogenesis of AD.

**Fig. 4:**
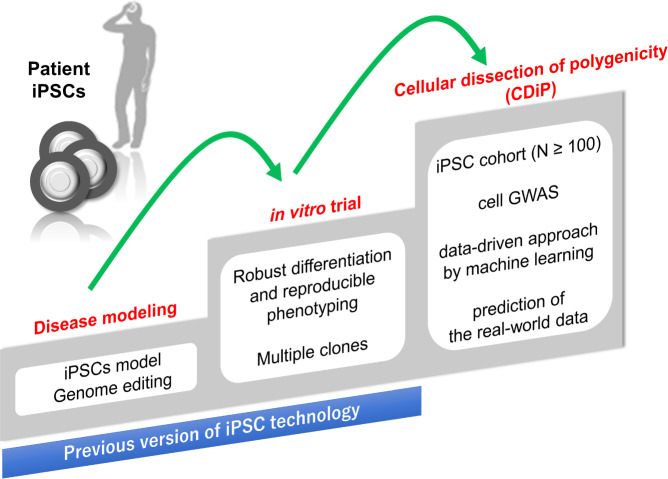
Cellular dissection of polygenicity (CDiP) technology as a novel approach to understanding genetic backgrounds at a cellular level

As shown in the example, cell GWAS is expected to generate information on new genes related to the disorders one after another as many as the number of cell types and phenotypes. Using the substantial data obtained in the iPSC cohort, we also worked on predicting the real-world data accumulated in clinical cohorts. The genetic background of SAD was regarded as polygenicity, and it was investigated by GWAS and other genetic cohorts. Efforts to predict the onset of AD by the use of genetic datasets have been conducted in the form of polygenic risk scores as in previous reports [1, 35, 36]. As expected, we succeeded in predicting Aβ deposits in the brain detected by positron emission tomography (PET) that altered the Aβ dose in cerebrospinal fluid in ADNI by machine learning with polygenic datasets found by cell GWAS. In the machine learning, we recruited a random forest classifier to predict whether Aβ deposition of a participant is positive or negative. Genotypes of ADNI participants were projected into the principal component space of iPSC cohort’s genotypes of the polygene detected by cell GWAS. Participants of ADNI cohorts were divided into a training set and a test set. A random forest classifier trained with the projected genotypes of the polygenes and covariates (age, sex, APOE genotype) of a training set demonstrated significantly improved classification performance in a test set compared with that trained only with the covariates. Potential evaluation of the direction of the principal component critical for the prediction may identify core polygenes for AD onset prediction. Alternatively, principal components analysis, or other dimensionality reduction methods with sparse constraints, might find more interpretable space of cell GWAS-derived polygenes. In addition, a similar approach may be potentially applicable to the prediction of other features of clinical cohorts such as onset age. In any event, we could finally establish an iPSC cohort and cell GWAS, and propose the concept of “Cellular dissection of polygenicity (CDiP)” to reconstruct the real-world data by using data-driven approach of iPSC-based datasets (Fig. 3). We believe that CDiP technology based on the iPSC cohort and cell GWAS is a novel version of genetic analysis and a prediction method for clinical events by iPSCs (Fig. 4) and will open a new era of investigating genetics for disorders.

## Conclusion

More than 15 years have passed since the advent of iPSCs, and now it is possible to conduct iPSC data-driven research to understand the genetics of brain disorders. Reversely, based on the results, iPSC-based disease models enable us to understand the pathological phenotypes originating from genetic backgrounds of AD, and to identify drug candidates with estimations of responsiveness. We look forward to a future where CDiP will contribute to reprogramming a future where AD is precisely predicted, avoided, and eradicated.
